# Discrete Homogeneous and Non-Homogeneous Markov Chains Enhance Predictive Modelling for Dairy Cow Diseases

**DOI:** 10.3390/ani14172542

**Published:** 2024-09-01

**Authors:** Jan Saro, Jaromir Ducháček, Helena Brožová, Luděk Stádník, Petra Bláhová, Tereza Horáková, Robert Hlavatý

**Affiliations:** 1 Department of Systems Engineering, Faculty of Economics and Management, Czech University of Life Sciences Prague, Kamycka 129, Suchdol, 165 00 Prague, Czech Republic; brozova@pef.czu.cz (H.B.); blahovap@pef.czu.cz (P.B.); horakovat@pef.czu.cz (T.H.); hlavaty@pef.czu.cz (R.H.); 2 Department of Animal Science, Faculty of Agrobiology, Food and Natural Resources, Czech University of Life Sciences Prague, Kamycka 129, Suchdol, 165 00 Prague, Czech Republic; duchacek@af.czu.cz (J.D.); stadnik@af.czu.cz (L.S.)

**Keywords:** dairy cows, herd health status, dairy diseases, Markov chains, predictive model, decision support systems

## Abstract

**Simple Summary:**

Managing cow diseases effectively remains a major challenge in dairy farming. Our study introduces a simple model for predicting dairy cow diseases. To develop this model, we used categorized data and Markov chains to select the best prediction model based on minimal error distance. The results show that our model is not only highly accurate and reliable but also easy to use, even in low-tech farms. Our methodological approach can capture various data structures in different volumes and qualities, demonstrating its versatility and adaptability to a wide range of herd sizes. This universal applicability enables us to evaluate entire herds, regardless of size. Furthermore, while each farm records diseases differently, our model can accommodate these variations. As such, this model may help dairy farmers manage herd health, predict antibiotic costs, and plan farming strategies.

**Abstract:**

Modelling and predicting dairy cow diseases empowers farmers with valuable information for herd health management, thereby decreasing costs and increasing profits. For this purpose, predictive models were developed based on machine learning algorithms. However, machine-learning based approaches require the development of a specific model for each disease, and their consistency is limited by low farm data availability. To overcome this lack of complete and accurate data, we developed a predictive model based on discrete Homogeneous and Non-homogeneous Markov chains. After aggregating data into categories, we developed a method for defining the adequate number of Markov chain states. Subsequently, we selected the best prediction model through Chebyshev distance minimization. For 14 of 19 diseases, less than 15% maximum differences were measured between the last month of actual and predicted disease data. This model can be easily implemented in low-tech dairy farms to project costs with antibiotics and other treatments. Furthermore, the model’s adaptability allows it to be extended to other disease types or conditions with minimal adjustments. Therefore, including this predictive model for dairy cow diseases in decision support systems may enhance herd health management and streamline the design of evidence-based farming strategies.

## 1. Introduction

Dairy farming improves human welfare globally. Directly or indirectly, the dairy sector employs approximately 240 million people and provides a livelihood for up to one billion people worldwide. Furthermore, milk production promotes female empowerment [1], as well as sustainable production and consumption patterns [2] and water and sanitation management [3], in line with sustainable development goals (SDGs) 10, 12 and 6, respectively. In turn, increasing dairy intake reduces healthcare costs [4] and inequalities in food security and nutrition [5]. The need for sustainable livestock production in response to challenges is using a farm animal algorithm in order to address the population increase and avoid food problems in the future [6].

Milk production and reproduction are influenced by a multitude of factors that complement each other [7,8] and can be useful in diagnosing various problems and diseases. In dairy cattle, a wide range of diseases occur, from reproductive tract issues to problems with the mammary gland [9,10] and limbs [11], and even metabolic diseases [12] affecting the general resilience of dairy cows [13]. Most of these diseases have significant economic implications due to reduced milk yield and, for example, the necessity for early culling of dairy cows. Dairy cow diseases considerably decrease farm productivity [14]. In addition to adversely affecting animal welfare by causing pain and discomfort [15], dairy cow diseases such as digital dermatitis decrease milk yield [16] and lead to fertility problems [17]. Making matters worse, diseases like mastitis can affect milk quality and safety, posing risks to human health [18]. Due to increased veterinary costs and loss of livestock, these diseases financially strain dairy farms, which incur high economic losses [19]. Minimizing such economic losses may require a one-health approach to dairy production [20], including research on disease prevention and modelling.

Modelling and predicting dairy cow diseases using precision livestock farming approaches [21] and/or enhancing cattle production and management through convolutional neural networks [22] provides dairy farmers with valuable information for effective herd health management through strategies specifically designed to tackle each disease individually [23,24]. Projecting disease occurrences enables dairy farmers to improve animal health [25]. As a result, dairy farmers not only observe a positive impact on animal health [26] but also increase their profitability [27], primarily by decreasing costs with antibiotics [28].

Predictive models for dairy cow diseases were developed based on several research directions. Dairy diseases can be detected with wearable precision dairy technologies [29,30] and processed at the disease with machine learning [31]. In practice, machine learning algorithms were applied to project lameness [32] and combined with sensor data to predict mastitis [33]. However, machine learning-based approaches require developing a specific model for each disease. Conversely, other models can predict diseases at the herd level by regularly collecting herd summary data and applying parametric and nonparametric approaches to forecast herd health conditions, but not at the disease level [34]. Therefore, developing a model for simultaneously predicting several diseases may demand alternative approaches, such as Markov chains.

Markov chains have already been applied for cow behavior analysis and calving time prediction [35]. A Markov chain model with two states, shedding and non-shedding, was developed to analyze Listeria monocytogenes fecal shedding in dairy cattle [36]. Furthermore, Hidden Markov models were used to project healthy or diseased states based on monthly somatic cell scores of dairy cows with or without clinical mastitis [37] and to detect lameness in image records of cow movements [38]. However, as in the machine learning studies described above, low data availability limits the consistency of these models [39]. Nevertheless, a Markov chain model was integrated with a daily dynamic programming model to assess the effect of reproductive performance on dairy cattle herd value [40].

The present study aims at leveraging Markov chains to effectively model and predict the progression and occurrence of dairy cow diseases during lactation towards improving decision-making [41,42,43], and farm management about herd health and cutting costs [44].

## 2. Materials and Methods

### 2.1. Data Description

A dataset of 36 diseases was collected for 750 dairy cows of a herd housed in a farm located in the Czech Republic during the six-year period from 1 January 2018 to 7 December 2023, totaling 2167 days. This dataset contained the count of occurrences of each dairy cow disease monitored daily during the study period.

The data were continuously collected by the dairy farm’s zootechnician and subsequently processed using Python scripts. During the data pre-processing stage, it was essential to carry out extensive data cleaning, including the standardization of data formats and the removal of duplicate entries. These steps were critical to ensure the reliability and consistency of the dataset used in our analysis.

Table 1 presents a statistical summary of these disease data.

The data outlined in Table 1 highlight the low occurrence of most diseases in this dairy farm.

### 2.2. Statistical Methods

To assess differences in dairy cow disease occurrences during the study period, we performed the nonparametric Kruskal–Wallis test using time series data for each disease. Based on the results from this test, we identified significant quarterly differences in variables for each disease (the significance level for this study is set to 5%).

### 2.3. Criteria for Model Selection

Initially, we analyzed the data to identify frequent diseases. For such diseases, we applied the Markov Chain model; otherwise, we used the Elementary probability model.

For each disease i=1,2,…,D, quarter q=1,2,…,Q, and day t=1,2,…,N, two markers are calculated, namely $O1\left(i,q\right)$ and $O2\left(i\right)$.

The marker $O1\left(i,q\right)$ is equal to zero when disease i does not occur in quarter q; otherwise, the marker is equal to one. The marker $O1\left(i,q\right)$ is calculated according to the following formula:

(1) $$O1\left(i,q\right)=sgn\left(\sum_{t\in Q_{q}}d\left(i,t\right)\right)$$

where $d\left(i,t\right)$ is the number of occurrences of dairy disease i on day t*,* the set $Q_{q}$ consists of all days in quarter q.

The marker $O2\left(i\right)$ expresses the number of days disease i occurs throughout the monitoring period. This marker is calculated according to the following formula:

(2) $$O2\left(i\right)=\sum_{t\in N}sgn\left(d\left(i,t\right)\right)$$

where $d\left(i,t\right)$ is the number of occurrences of dairy disease i on day t and N represents the set of all monitoring days.

The decision to use the Elementary probability model or the Markov Chain model is made based on the relative number of quarters i and on the relative number of days i during which the disease occurs. Two indexes are calculated $F1\left(i\right)$ and $F2\left(i\right)$ as follows:

(3) $$F1\left(i\right)=\frac{\sum_{k=1}^{n}O1\left(i,k\right)}{Q}$$

where Q is number of monitored quarters.

(4) $$F2\left(i\right)=\frac{O2\left(i\right)}{N}$$

where N is total number of monitored days.

The following rule for model selection is applied:

(5) $$\begin{array}{c} If\;F1\left(i\right)>0.5\;and\;F2\left(i\right)>0.01,\;then\;the\;Markov\;Chain\;model\;is\;used;\\ otherwise\;\left(if\;F1\left(i\right)\leq 0.5\;or\;F2\left(i\right)\leq 0.01\right),\;the\;Elementary\;Probability\;model\;is\;used. \end{array}$$

### 2.4. Description of the Model

#### 2.4.1. Classical Probabilistic Model

**Figure 1:**
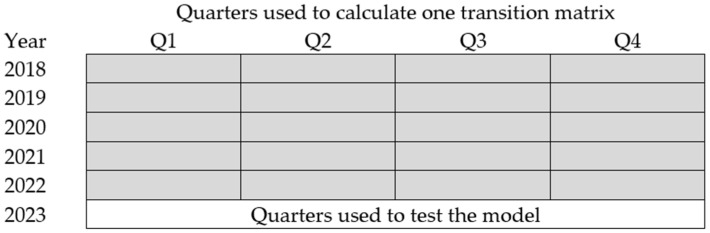
Quarters used across multiple years to calculate a single transition matrix for the HMC, with the final set of quarters in 2023 used for model testing.

**Figure 2:**
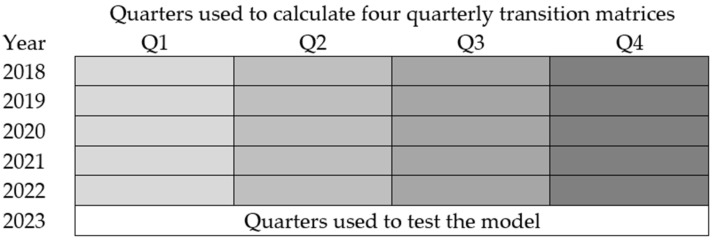
Grouping of quarters across years used to calculate four quarterly transition matrices for the NHMC, with the final set of quarters in 2023 used for model testing.

The classical probability model is chosen if a rare disease occurrence is assumed based on the Formula (5). Two states are then considered: 0—the disease does not occur, and 1—the disease occurs. The probability $\hat{p}\left(i,1\right)$ of the occurrence of the disease i is calculated as a relative frequency using the following formula:

(6) $$\hat{p}\left(i,1\right)=\frac{\sum_{t=1}^{N-T}d(i,t)}{N-T}$$

where $d\left(i,t\right)$ is the number of occurrences of dairy disease i on day t, N is the total number of days, and T is the number of the last days used to test the prediction (Figure 1 and Figure 2).

Accordingly, the probability $\hat{p}\left(i,0\right)$ of non-occurrence of disease i is calculated using the following formula:

(7) $$\hat{p}\left(i,0\right)=1-\hat{p}\left(i,1\right)$$

The accuracy of this model is tested by comparing its results with real data using Chebyshev distance, which is particularly suitable for highlighting the maximum deviation between predicted and actual values, thereby providing a clear measure of the model’s worst-case error performance.

#### 2.4.2. Discrete Markov Chain Model

A Markov chain is a stochastic process that models the probability of transition from one state to another, where the next state depends only on the current state and not on the sequence of events that preceded it (the “memoryless” property). If the Discrete Markov chain model was selected in the previous phase to predict disease occurrence based on Formula (5), discrete Homogeneous (HMC) or Non-homogeneous (NHMC) Markov chain model accuracy is tested using Chebyshev distance. For this purpose, the Markov chain states are defined first, and then either the Transition matrix is calculated for the HMC model or the four Transition matrices are calculated for the NHMC model and each season. After the predictions, the accuracy of the model is calculated using Chebyshev distance to compare the results with real data.

To clarify the differences between the models, a Homogeneous Markov Chain (HMC) assumes that the transition probabilities remain constant over quarters, which simplifies the modeling process when disease occurrence patterns are relatively stable throughout the year. On the other hand, a Non-homogeneous Markov Chain (NHMC) allows transition probabilities to vary over quarters, capturing temporal or seasonal variations in disease dynamics. This flexibility in the NHMC model is crucial for scenarios where disease progression is influenced by seasonal factors, making it a more suitable choice when the data suggest periodic changes in disease occurrence.

Step 1—Definition of the states of the Markov chain model

The states of the Markov chain model are defined as the number of dairy cows affected by the disease per day. All states form the set $\left\{0,1,2,\ldots ,M\right\}$, where M is the number of dairy cows, 0 means that no dairy cow is affected by the disease per day, and M means that all dairy cows are affected by the disease per day. However, only a smaller number k≤M of cows is affected usually. Therefore, the real set of states of disease i is

(8) $$\begin{array}{ll} S(i) & =\left\{s_{0},s_{1},\ldots ,s_{K-1},s_{K}\right\}\\ & =\left\{0,\;1,\ldots ,\underset{t=1,\ldots ,N-T}{\max}\left(d\left(i,t\right)\right)-1,\underset{t=1,\ldots ,N-T}{\max}\left(d\left(i,t\right)\right)\right\} \end{array}$$

where $d\left(i,t\right)$ is the number of occurrences of dairy cow disease i on day t.

If the probability of states referring to the highest number of disease occurrences per day is very low, the following subset $S_{R}(i)$ of the set of states S(i) of the Markov chain model is used:

(9) $$S_{R}(i)\subseteq S(i)$$

where 0, 1, …, R are elements of $S_{R}(i),\;R\leq K$, and state R aggregates all other $\left\{s_{R},s_{R+1},\ldots ,s_{K}\right\}$ states.

Step 2—Homogenous Markov Chain

Assuming the homogeneity of the process during the monitoring period, we first determine the Transition matrices for all possible numbers of states R (Figure 1). For each disease i, the transition probabilities are calculated using the following formula:

(10) $$\begin{array}{c} P_{R}\left(i\right)=\left(\begin{array}{cccc} p_{R}\left(i,1,1\right) & p_{R}\left(i,1,2\right) & \ldots & p_{R}\left(i,1,R\right)\\ p_{R}\left(i,2,1\right) & \ddots & & \vdots \\ \vdots & & & \\ p_{R}\left(i,R,1\right) & \ldots & & p_{R}\left(i,R,R\right) \end{array}\right)\\ \mathrm{where}\;p_{R}\left(i,a,b\right)=\frac{\sum_{t=1}^{N-T}c_{ab}\left(i,t\right)}{\sum_{b\in S_{R}\left(i\right)}\sum_{t=1}^{N-T}c_{ab}\left(i,t\right)} \end{array}$$

where $p_{R}\left(i,a,b\right),a,b=1,2,\ldots ,R,$ is the probability of the transition from state a sick dairy cows to state b sick dairy cows, N is the count of all days, T is the length of the predicted period, and $c_{ab}\left(i,t\right)$ is equal to either 1 if the transition from state a to state b occurs in time t or 0 otherwise.

At the end, the transition matrix is calculated for all reasonable R for which $\left\lfloor K/2\right\rfloor \leq R\leq K$ because a smaller number of states would not describe the numbers of sick cows well enough.

State probabilities for each disease i=1,…,N are predicted as follows:

(11) $${\hat{p}}_{R}^{T}\left(i\right)=p_{init}\cdot P_{R}^{T}\left(i\right)=p_{init}\cdot P_{R}\left(i\right)\cdot P_{R}\left(i\right)\cdot \ldots \cdot P_{R}\left(i\right)$$

where ${\hat{p}}_{R}^{T}(i)=({\hat{p}}_{R}\left(i,0\right),{\hat{p}}_{R}\left(i,1\right),\ldots ,{\hat{p}}_{R}\left(i,R\right))$ is the predicted distribution of states probabilities in predicted period T, $p_{init}$ is vector of initial state probabilities with dimension R with all zeros, except the state describing the last count of occurrences of disease i, where its value is 1, $P_{R}^{T}\left(i\right)=P_{R}\left(i\right)\cdot P_{R}\left(i\right)\cdot \ldots \cdot P_{R}\left(i\right)$ is the transition matrix from time N−T+1 to N, i.e., T-th power of matrix $P_{R}\left(i\right)$, and T is the length of the predicted period.

The best value $R^{*}$ is selected based on Chebyshev distance minimization to identify the best predictive accuracy:

(12) $$R^{*}=arg\underset{R=\left\lfloor K/2\right\rfloor ,\ldots ,K}{\min}\left\{\underset{j=1,\ldots ,R}{\max}\left(\left|{\hat{p}}_{r}^{T}\left(i,j\right)-\frac{\sum_{t=N-T+1}^{N}c_{j}\left(i,t\right)}{T}\right|\right)\right\}$$

where ${\hat{p}}_{r}^{T}(i,j)$ is a j-th element of the vector ${\hat{p}}_{R}^{T}\left(i\right),$ i.e., predicted probability of state j, and $c_{j}\left(i,t\right)$ is equal to either 1 if j dairy cows were sick with disease i in time t or 0 otherwise.

Based on predicted state probabilities the mean value of disease occurrences per day can be calculated as:

(13) $$\hat{m}\left(i\right)={\hat{p}}_{R}^{T}\left(i\right)\cdot {\left(0,1,2,\ldots ,R^{*}\right)}^{\prime }$$

The mean value of disease occurrences per day, $\hat{m}\left(i\right)$, is calculated as the scalar product of two vectors: the vector of predicted state probabilities ${\hat{p}}_{R}^{T}\left(i\right)$ and the transposed vector of possible disease occurrence states ${\left(0,1,2,\ldots ,R^{*}\right)}^{\prime }$.

Step 3—Non-Homogenous Markov Chain

If the quarter data show a non-homogeneous process, all four quarterly transition matrices must be calculated, that is, one for each predicted quarter (Figure 2).

These four transition matrices are then tested using Formulas (10)–(12) regarding the split time span.

### 2.5. Calculation of the Prediction Model for Dairy Cow Diseases

**Figure 3:**
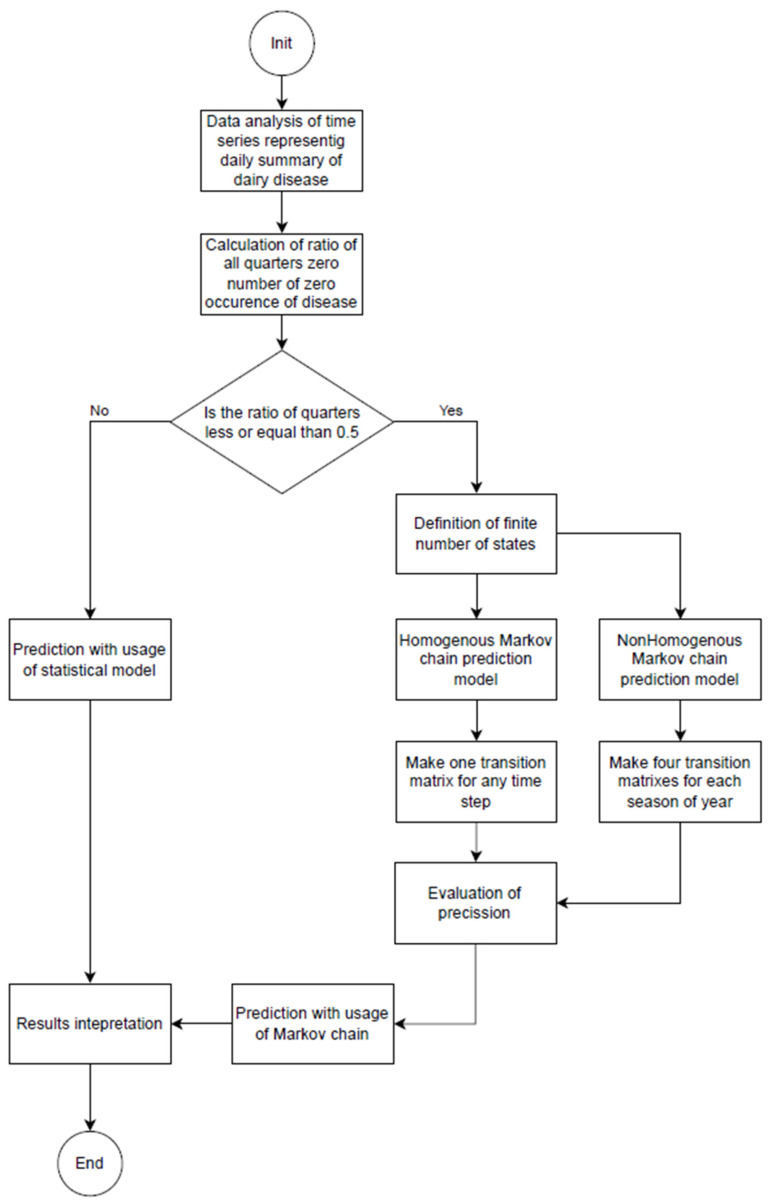
Flow diagram of the calculation of the prediction model for dairy cow diseases.

Figure 3 shows a flow diagram of the individual steps taken in the process of predicting the number of sick cows in a specific period.

Markov chain model was implemented, calculated, and tested using Python programming language.

## 3. Results

In this study, we used three mathematical models, namely Elementary Probability and discrete HMC and NHMC models, to predict the probability distribution of dairy cow diseases in the next one and two months.

### 3.1. Rare Diseases

The results of the prediction of disease occurrences in the next month, assuming that $F1\left(i\right)\leq 0.5\;or\;F2\left(i\right)\leq 0.01$, are presented in Table 2. The predicted probabilities of disease occurrences are computed using the Elementary probability model expressed by Formulas (6) and (7).

### 3.2. Prevalent Diseases

The HMC model was first applied to predict the state probabilities in the next one (model HMC30) and two (model HMC60) months. The results of the prediction of disease occurrences in the next months, assuming that $F1\left(i\right)>0.5\;and\;F2\left(i\right)>0.01,$ are presented in Table 3.

For the next month, the mean Chebyshev distance was 0.132, and the median value was 0.104. For the next two months, the predictive performance of the Markov chain model reached a mean Chebyshev distance of 0.189, with a median value of 0.2. The maximum deviations of Chebyshev distance were observed when predicting the occurrence of ‘Necrobacillosis’ and ‘Mastitis LF’ using the HMC model.

As shown in Appendix A, the mean value of the first state (healthy herd) across all records was approximately 0.761 for one month and 0.761 for two months. These values highlight the healthy state of the dairy herd.

The NHMC model was then applied to predict the probability distribution for the next one and two months. The NHMC results are presented in Table 4. Transition matrices were calculated separately for each quarter. The mean Chebyshev distance was 0.12, and the median value was 0.088 for one month of prediction. The predictive performance of the NHMC model for the next two months reached a mean Chebyshev distance of 0.101, with a median value of 0.074. As shown in Appendix A, the mean value of the first state was approximately 0.731 for the next month and 0.732 for the next two months, across all records. These results demonstrate the healthy state of the dairy herd.

The results enabled us to compare two approaches, namely the HMC and the NHMC models, to assess their accuracy using Chebyshev distance. For one and two months, the predictive accuracy of the HMC model was 0.132 and 0.189, respectively. In turn, for the same intervals, the predictive accuracy of the NHMC model was 0.144 and 0.101, respectively. Thus, HMC is more accurate than NHMC. For all diseases, the mean probability of the non-occurrence of the disease was higher than 79%.

### 3.3. Analysis of the Results

In this section, we analyze the results from the predictive model for diseases Metabolic problems, Mastitis RB and Reproduction problems, respectively.

### 3.4. Metabolic Problems

**Figure 4:**
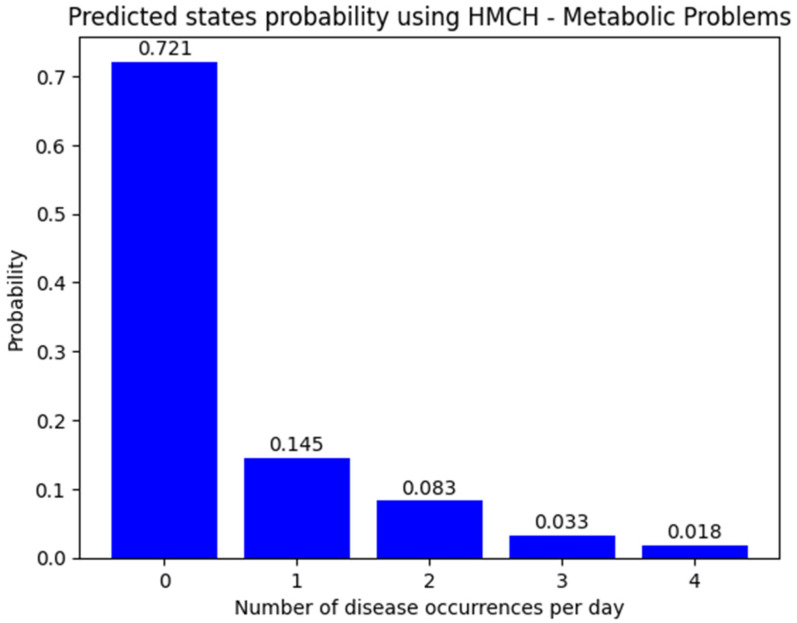
Predicted probability distribution of metabolic problems—homogenous Markov chain model for next 30 days.

The mean value of the expected number of occurrences per day is 0.482, according to the HMC model. Even the histogram (Figure 4) of the probability of the number of metabolic problems shows that the state of no disease occurs on more than 72% of the days and the result accuracy of the HMC model has 0.05 measured by the Chebyshev distance.

### 3.5. Mastitis RB

**Figure 5:**
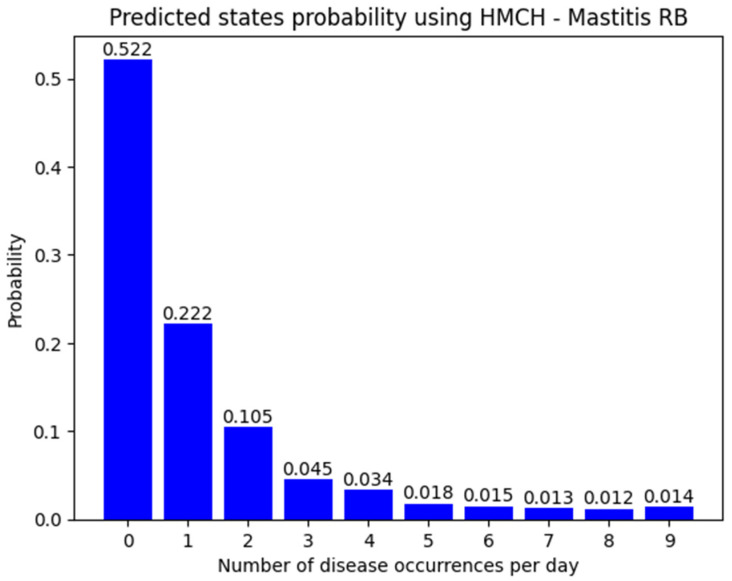
Predicted state probability using HMCH of Mastitis RB for next 30 days.

The expected mean value of Mastitis RB disease occurrences per day is 1.196. According to the histogram (Figure 5) of probabilities of the number of sick dairy cows shown in Figure 5, the state of no disease occurs in less than 52% of the days rounded on decimals.

### 3.6. Reproduction Problems

**Figure 6:**
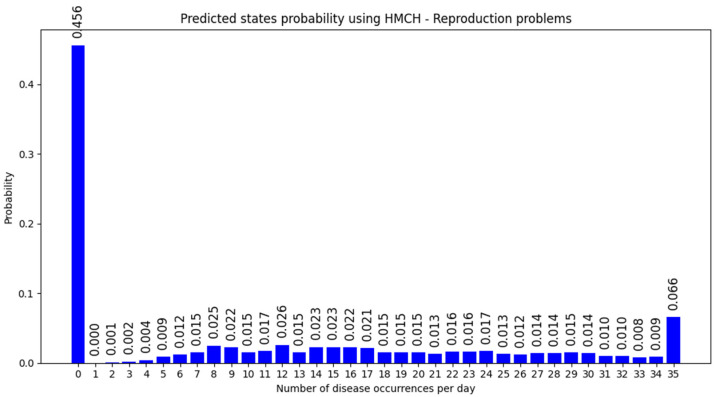
Predicted state probability using HMCH of Reproduction problems for next 30 days.

The expected number of these diseases was 10.892 per day predicted by the homogenous Markov chain model for the next 30 days with an accuracy of 0.145 measured by Chebyshev distance. According to the histogram shown in Figure 6 of the probabilities of the number of dairy disease occurrences per day, the state of no disease occurs in less than 46% of the days. These results highlight the need to prepare for a relatively high number of 10 sick dairy cows per day.

## 4. Discussion

To effectively model and predict the progression and occurrence of dairy cow diseases during lactation, we selected Markov chain models because the number of dairy cows with a disease in a forecasted period depends on the number of cows with this disease in the previous period [45]. Based on our data analysis and on the accuracy of the results, we further selected HMC rather than NHMC. The HMC model can be used to support the decision-making process in estimating the number of individual diseases, monitoring the development of herd health status and determining the appropriate intensity of veterinary services in dairy farms.

Our HMC model is applicable as a prediction tool for dairy cow diseases in a wide range of dairy farms, regardless of their technological level [46]. As a predictive component, this model may also be integrated into a decision support system to improve our ability to predict and manage the health conditions of dairy herds [47], in addition to supporting effective decision-making by predicting potential health outcomes. Leveraging advanced statistical methods for short-term forecasting, this new methodological approach can significantly enhance decision support by capturing various data structures in different volumes and qualities. In addition, this model can be applied to herds of different sizes worldwide to evaluate entire herds from a specific number of animals. Thus, our model enables proactive dairy health management strategies.

During its use, the Markov chain must be updated, which entails updating the values of the matrix of transition probabilities either immediately with each forecast query or after a predetermined period. Because the former approach has the disadvantage of overestimating even instantaneous fluctuations, the latter seems more appropriate. However, this approach requires moving the time window. To this end, the recommended length of forecasts is one-fifth of the length of the time series, but predictions over longer periods are also feasible, up to a quarter.

The accuracy of our HMC and NHMC models in predicting the number of diseases of dairy cows did not significantly differ from that of a similar study using an NHMC model in different time periods [48]. However, nonhomogeneous Markov chain prediction [49] using appropriate intervals is a feasible alternative for further research and experiments with disease time series aimed at detecting sub-trends.

### Practical Use

Our model may be used as a Markov Chain Decision Process (MCDP) to project individual diseases, thereby assessing veterinarian needs in dairy farms. Based on two different actions, our model enables us to measure differences in two mean values and to increase health state probability. As a prediction tool for dairy cow diseases, this framework is applicable to a wide range of dairy farms, including low-tech farms [46]. Unlike precision livestock farming (PLF) applications, which often require substantial investment in technological infrastructure and real-time sensor data, our model provides a statistically robust alternative that remains accessible and effective for farms with limited resources or lower levels of technological advancement. This makes it particularly suitable for low-tech or smaller-scale operations, where the implementation of PLF systems may be cost-prohibitive. As a predictive component, its incorporation into a Dairy Disease Decision Support System (DSS) may enhance dairy herd health prediction and management [47], effectively supporting decision-making by forecasting potential health outcomes and, therefore, enabling proactive management strategies. This novel approach to statistically leverage data to predict short-term trends supports decision-making processes.

## 5. Conclusions

Our Markov chain model is a promising tool for predicting the occurrence of dairy cow diseases in the next month. With practical adaptations, this model can be efficiently implemented in dairy farms for farmers to gather useful information for farm health management. One of the key advantages of the Markov chain model is its ability to provide accurate predictions even with limited or incomplete data, making it highly applicable in real-world farming conditions. This model can be incorporated into decision support systems for disease prognosis and strategy design in dairy farms to cut costs with antibiotics for individual diseases, monitor the quality of veterinary services and develop dairy health programs based on disease occurrence. Based on the achieved results, extending the design and development of new applications will be an objective for further research.

## Figures and Tables

**Table 1:** Basic statistics—summary data of occurrences of dairy cow disease over 5 years.

Diseases	Min Occurrence	Max Occurrence	Sum of All Occurrences	Mean Occurrence	SD	F1	F2
Abscess	0	1.0	1.0	0.000	0.021	0.958	0.000
Acidosis	0	1.0	3.0	0.001	0.037	0.917	0.001
Tympani	0	3.0	4.0	0.002	0.068	0.917	0.001
Bleeding	0	2.0	5.0	0.002	0.057	0.875	0.002
Dermatitis	0	2.0	6.0	0.003	0.061	0.875	0.002
Pneumonia	0	2.0	7.0	0.003	0.071	0.958	0.002
Udder Edema	0	1.0	7.0	0.003	0.057	0.875	0.003
Pain	0	1.0	11.0	0.005	0.071	0.833	0.005
Nerve Damage	0	2.0	14.0	0.006	0.091	0.708	0.006
Jaw Edema	0	2.0	15.0	0.007	0.098	0.875	0.006
Postpartum Sepsis	0	4.0	17.0	0.008	0.130	0.875	0.005
Damaged Teat	0	2.0	21.0	0.010	0.103	0.708	0.009
Torn During Birth	0	3.0	25.0	0.012	0.127	0.667	0.010
Digestive Troubles	0	4.0	28.0	0.013	0.160	0.833	0.008
Abomasal Dilatation	0	3.0	32.0	0.015	0.157	0.625	0.010
Peritonitis	0	2	35	0.016	0.156	0.792	0.012
Mastitis	0	5	39	0.018	0.238	0.708	0.008
Eye Injury	0	3	51	0.024	0.190	0.458	0.018
High somatic in milk	0	6	70	0.032	0.359	0.875	0.011
Postpartum hypocalcemia	0	4	97	0.045	0.331	0.333	0.020
Phlegmon	0	4	159	0.073	0.346	0.208	0.052
Respiration	0	4	162	0.075	0.350	0.375	0.052
Laminitis	0	4	226	0.104	0.415	0.083	0.074
Retained placenta	0	3	287	0.132	0.403	0.042	0.111
Limb Edema	0	3	330	0.152	0.456	0.042	0.116
Diarrhea	0	7	372	0.172	0.604	0.417	0.102
High temperature after calving	0	5	541	0.250	0.604	0.000	0.180
Uterus	0	14	582	0.269	1.111	0.167	0.096
High temperature	0	5	821	0.379	0.798	0.000	0.235
Endometritis	0	15	834	0.385	1.369	0.208	0.111
Necrobacillosis	0	6	996	0.460	0.877	0.000	0.287
Metabolic problems	0	6	1047	0.483	0.927	0.000	0.278
Mastitis RB	0	16	2643	1.220	2.007	0.000	0.482
Mastitis RF	0	19	2806	1.295	2.211	0.000	0.493
Mastitis LB	0	18	3103	1.432	2.363	0.042	0.499
Mastitis LF	0	23	6851	3.162	3.488	0.000	0.800

Diseases—names of dairy diseases; Min Occurrence—minimal daily occurrence of dairy disease; Max Occurrence—minimal daily occurrence of dairy disease; Sum of all Occurrences—sum of all occurrences of dairy disease per the whole time period; Mean Occurrence—mean value of occurrences of dairy disease per the whole time period; SD—standard deviation of dairy occurrences per the whole time period; F1—the relative number of quarters i during which the disease occurs (described below); F2—a relative number of occurrence of disease per a total number of monitored days (described below).

**Table 2:** Basic probability model for predicting rare diseases that do not meet the selection criteria.

Diseases	Total Sum of Disease Occurrence	$\hat{p}\left(i,0\right)$	$\hat{p}\left(i,1\right)$
Abscess	1	1	0.000
Acidosis	3	0.999	0.001
Tympani	4	0.998	0.002
Bleeding	5	0.998	0.002
Dermatitis	6	0.997	0.003
Pneumonia	7	0.997	0.003
Udder Edema	7	0.997	0.003
Pain	11	0.996	0.004
Nerve Damage	14	0.994	0.006
Jaw Edema	15	0.993	0.007
Postpartum Sepsis	17	0.992	0.008
Damaged Teat	21	0.99	0.010
Torn During Birth	25	0.988	0.012
Digestive Troubles	28	0.987	0.013
Abomasal Dilatation	32	0.985	0.015
Peritonitis	35	0.984	0.016
Mastitis	39	0.982	0.018
High somatic	70	0.968	0.032

Diseases—names of dairy diseases; Total sum of disease occurrence; $\hat{p}\left(i,0\right)$—a predicted probability of non-occurrence of disease i; $\hat{p}\left(i,1\right)$—a predicted probability of an occurrence of disease i.

**Table 3:** Basic probability model for predicting prevalent diseases that meet the selection criteria.

Diseases	Min Number of States	Max Number of States	HMC30 Opt. $\mathrm{Number}\;\mathrm{of}\;\mathrm{States}\;R^{*}$	HMC30 Chebyshev Distance	HMC30 Mean Value of Dairy Disease Occurrences	HMC60 $\mathrm{Opt}.\;\mathrm{Number}\;\mathrm{of}\;\mathrm{States}\;R^{*}$	HMC60 Chebyshev Distance	HMC60 Mean Value of Dairy Disease Occurrences
Eye Injury	2	4	3	0.019	0.023	4	0.250	0.025
Postpartum hypocalcemia	2	5	5	0.021	0.046	4	0.250	0.045
Phlegmon	2	5	4	0.052	0.073	4	0.250	0.074
Respiration	2	5	5	0.032	0.076	4	0.250	0.076
Laminitis	2	5	4	0.019	0.103	4	0.250	0.098
Retained placenta	2	4	3	0.025	0.131	3	0.333	0.129
Limb Edema	2	4	4	0.031	0.153	3	0.333	0.151
Diarrhea	2	8	8	0.104	0.177	5	0.200	0.167
High temperature after calving	2	6	4	0.149	0.248	4	0.250	0.252
Uterus	2	15	9	0.092	0.257	9	0.111	0.263
High temperature	2	6	6	0.168	0.381	4	0.250	0.357
Endometritis	2	16	9	0.134	0.378	9	0.111	0.371
Necrobacillosis	2	7	5	0.452	0.439	5	0.200	0.435
Metabolic problems	2	7	5	0.050	0.482	5	0.200	0.467
Mastitis RB	2	17	10	0.222	1.196	10	0.100	1.214
Mastitis RF	2	20	17	0.193	1.293	16	0.063	1.301
Mastitis LB	2	19	11	0.121	1.386	11	0.091	1.4
Mastitis LF	2	24	22	0.472	3.154	13	0.077	3.105
Reproduction problems	2	70	36	0.145	10.892	36	0.028	10.895

Diseases—names of dairy diseases; Min number of states—minimal number of states of Markov chain; Max number of states—maximum number of states of Markov chain; HMC30 Opt. number of states $R^{*}$—Optimal number of states calculated for Homogenous Markov chain model for next 30 days; HMC30 Opt. number of states Chebyshev distance—Chebyshev distance for an Optimal number of states calculated for Homogenous Markov chain model for next 30 days; HMC30 Mean value of dairy disease occurrences—Mean value calculated for next 30 days of dairy disease occurrence; HMC60 Opt. number of states $R^{*}$—Optimal number of states calculated for Homogenous Markov chain model for next 60 days; HMC60 Opt. number of states Chebyshev distance—Chebyshev distance for an Optimal number of states calculated for Homogenous Markov chain model for next 60 days; HMC60 Mean value of dairy disease occurrences—Mean value calculated for next 60 days of dairy disease occurrence.

**Table 4:** Results of non-homogenous Markov chains.

Diseases	Min Number of States	Max Number of States	NHMC30 Opt. $\mathrm{Number}\;\mathrm{of}\;\mathrm{States}\;R^{*}$	NHMC30 Chebyshev Distance	NHMC30 Mean Value of Dairy Disease Occurrences	NHMC60 $\mathrm{Opt}.\;\mathrm{Number}\;\mathrm{of}\;\mathrm{States}\;R^{*}$	NHMC60 Chebyshev Distance	NHMC60 Mean Value of Dairy Disease Occurrences
Eye Injury	2	4	4	0.053	0.117	4	0.055	0.121
Postpartum hypocalcemia	2	5	4	0.100	0.281	4	0.103	0.289
Phlegmon	2	5	5	0.144	0.389	5	0.056	0.385
Respiration	2	5	4	0.045	0.157	4	0.065	0.163
Laminitis	2	5	4	0.027	0.143	5	0.034	0.183
Retained placenta	2	4	3	0.025	0.138	4	0.013	0.199
Limb Edema	2	4	3	0.041	0.176	3	0.074	0.182
Diarrhea	2	8	5	0.175	0.397	5	0.054	0.383
High temperature after calving	2	6	4	0.147	0.248	4	0.137	0.258
Uterus	2	15	9	0.083	0.47	9	0.036	0.477
High temperature	2	6	6	0.176	0.469	4	0.171	0.41
Endometritis	2	16	9	0.128	1.105	9	0.042	1.134
Necrobacillosis	2	7	7	0.410	0.756	7	0.269	0.744
Metabolic problems	2	7	6	0.070	0.398	7	0.156	0.626
Mastitis RB	2	17	14	0.188	2.606	10	0.104	1.399
Mastitis RF	2	20	11	0.221	1.946	11	0.086	2.003
Mastitis LB	2	19	11	0.104	1.404	11	0.130	1.447
Mastitis LF	2	24	14	0.460	3.599	14	0.263	3.744
Reproduction problems	2	70	40	0.144	12.566	40	0.071	12.55

Diseases—names of dairy diseases; Min number of states—minimal number of states of Markov chain; Max number of states—maximum number of states of Markov chain; NHMC30 Opt. number of states $R^{*}$—Optimal number of states calculated for Non-Homogenous Markov chain model for next 30 days; NHMC30 Opt. number of states Chebyshev distance—Chebyshev distance for an Optimal number of states calculated for Non-Homogenous Markov chain model for next 30 days; NHMC30 Mean value of dairy disease occurrences—Mean value calculated for next 30 days of dairy disease occurrence; NHMC60 Opt. number of states $R^{*}$—Optimal number of states calculated for Non-Homogenous Markov chain model for next 60 days; NHMC60 Opt. number of states Chebyshev distance—Chebyshev distance for an Optimal number of states calculated for Non-Homogenous Markov chain model for next 60 days; NHMC60 Mean value of dairy disease occurrences—Mean value calculated for next 60 days of dairy disease occurrence.

## Data Availability

The data presented in this study are available on request from the corresponding author.

## Appendix A

**Table A1:** Predicted probability distributions of states with homogeneous and non-homogeneous Markov chains.

Diseases	HMC 30 Optimal Number of States	HMC 30 Predicted Distribution	HMC 60 Optimal Number of States	HMC 60 Predicted Distribution	NHMC 30 Optimal Number of States	NHMC 30 Predicted Distribution	NHMC 60 Optimal Number of States	NHMC 60 Predicted Distribution
Eye Injury	3	[ 0.981 0.015 0.004 ]	4	[ 0.981 0.015 0.002 0.002 ]	4	[ 0.947 0.019 0.004 0.03 ]	4	[ 0.945 0.02 0.004 0.031 ]
Postpartum hypocalcemia	5	[ 0.979 0.004 0.01 0.006 0.001 ]	4	[ 0.979 0.004 0.01 0.007 ]	4	[ 0.9 0.007 0.005 0.088 ]	4	[ 0.897 0.007 0.006 0.09 ]
Laminitis	4	[ 0.926 0.05 0.019 0.005 ]	4	[ 0.928 0.05 0.018 0.004 ]	4	[ 0.906 0.057 0.025 0.012 ]	5	[ 0.901 0.055 0.022 0.004 0.018 ]
Retained placenta	3	[ 0.889 0.091 0.02 ]	3	[ 0.89 0.091 0.019 ]	3	[ 0.885 0.092 0.023 ]	4	[ 0.869 0.087 0.02 0.024 ]
Limb Edema	4	[ 0.884 0.082 0.031 0.003 ]	3	[ 0.883 0.083 0.034 ]	3	[ 0.865 0.094 0.041 ]	3	[ 0.859 0.1 0.041 ]
Diarrhea	8	[ 0.896 0.059 0.03 0.008 0.004 0.001 0.001 0.001 ]	5	[ 0.899 0.057 0.029 0.008 0.007 ]	5	[ 0.825 0.072 0.037 0.013 0.053 ]	5	[ 0.837 0.063 0.033 0.014 0.053 ]
High temperature after calving	4	[ 0.818 0.127 0.044 0.011 ]	4	[ 0.816 0.128 0.044 0.012 ]	4	[ 0.82 0.123 0.046 0.011 ]	4	[ 0.813 0.128 0.047 0.012 ]
Uterus	9	[ 0.905 0.041 0.018 0.007 0.01 0.005 0.007 0.004 0.003 ]	9	[ 0.905 0.041 0.017 0.007 0.01 0.005 0.007 0.004 0.004 ]	9	[ 0.875 0.05 0.017 0.004 0.011 0.002 0.002 0.004 0.035 ]	9	[ 0.875 0.051 0.014 0.004 0.012 0.002 0.002 0.004 0.036 ]
High temperature	6	[ 0.765 0.132 0.073 0.02 0.007 0.003 ]	4	[ 0.77 0.132 0.069 0.029 ]	6	[ 0.735 0.124 0.099 0.026 0.011 0.005 ]	4	[ 0.754 0.121 0.086 0.039 ]
Phlegmon	4	[ 0.948 0.034 0.015 0.003 ]	4	[ 0.949 0.032 0.015 0.004 ]	5	[ 0.856 0.049 0.02 0 0.075 ]	5	[ 0.861 0.043 0.021 0 0.075 ]
Respiration	5	[ 0.948 0.032 0.017 0.002 0.001 ]	4	[ 0.947 0.033 0.017 0.003 ]	4	[ 0.921 0.03 0.02 0.029 ]	4	[ 0.918 0.031 0.021 0.03 ]
Endometritis	9	[ 0.891 0.032 0.016 0.011 0.014 0.015 0.007 0.004 0.01 ]	9	[ 0.891 0.033 0.016 0.011 0.014 0.015 0.007 0.004 0.009 ]	9	[ 0.791 0.039 0.023 0.009 0.009 0.014 0.011 0.011 0.093 ]	9	[ 0.788 0.041 0.022 0.009 0.007 0.015 0.007 0.011 0.1 ]
Necrobacillosis	5	[ 0.719 0.173 0.073 0.02 0.015 ]	5	[ 0.721 0.172 0.073 0.019 0.015 ]	7	[ 0.677 0.164 0.067 0.022 0.013 0.002 0.055 ]	7	[ 0.686 0.159 0.065 0.019 0.013 0.002 0.056 ]
Metabolic problems	5	[ 0.721 0.145 0.083 0.033 0.018 ]	5	[ 0.726 0.143 0.084 0.032 0.015 ]	6	[ 0.776 0.117 0.063 0.027 0.011 0.006 ]	7	[ 0.756 0.103 0.061 0.021 0.008 0 0.051 ]
Mastitis RB	10	[ 0.522 0.222 0.105 0.045 0.034 0.018 0.015 0.013 0.012 0.014 ]	10	[ 0.52 0.222 0.104 0.045 0.035 0.018 0.015 0.014 0.012 0.015 ]	14	[ 0.488 0.154 0.09 0.046 0.02 0.019 0.019 0.023 0.02 0.007 0.006 0.001 0.001 0.106 ]	10	[ 0.543 0.171 0.096 0.054 0.023 0.023 0.022 0.027 0.023 0.018 ]
Mastitis RF	17	[ 0.507 0.221 0.112 0.057 0.036 0.017 0.013 0.008 0.007 0.006 0.003 0.004 0.001 0.002 0.001 0.002 0.003 ]	16	[ 0.506 0.219 0.112 0.057 0.036 0.018 0.014 0.008 0.008 0.007 0.003 0.004 0.001 0.002 0.001 0.004 ]	11	[ 0.479 0.191 0.107 0.047 0.025 0.015 0.019 0.008 0.008 0.019 0.082 ]	11	[ 0.48 0.182 0.106 0.049 0.024 0.016 0.021 0.008 0.008 0.02 0.086 ]
Mastitis LB	11	[ 0.503 0.213 0.1 0.05 0.046 0.021 0.017 0.013 0.011 0.007 0.019 ]	11	[ 0.504 0.211 0.099 0.05 0.046 0.021 0.017 0.013 0.012 0.007 0.02 ]	11	[ 0.504 0.229 0.101 0.035 0.04 0.012 0.016 0.015 0.013 0.007 0.028 ]	11	[ 0.508 0.22 0.099 0.035 0.04 0.013 0.017 0.016 0.014 0.007 0.031 ]
Mastitis LF	22	[ 0.195 0.212 0.153 0.121 0.077 0.056 0.042 0.033 0.025 0.019 0.018 0.012 0.009 0.01 0.005 0.006 0.003 0.001 0.002 0 0.001 0 ]	13	[ 0.194 0.209 0.151 0.123 0.078 0.057 0.043 0.033 0.025 0.019 0.018 0.012 0.038 ]	14	[ 0.206 0.206 0.138 0.109 0.061 0.049 0.038 0.028 0.019 0.019 0.016 0.019 0.011 0.081 ]	14	[ 0.204 0.191 0.13 0.115 0.064 0.052 0.04 0.03 0.02 0.02 0.016 0.02 0.012 0.086 ]
Reproduction problems	36	[ 0.456 0 0.001 0.002 0.004 0.009 0.012 0.015 0.025 0.022 0.015 0.017 0.026 0.015 0.023 0.023 0.022 0.021 0.015 0.015 0.015 0.013 0.016 0.016 0.017 0.013 0.012 0.014 0.014 0.015 0.014 0.01 0.01 0.008 0.009 0.066 ]	36	[ 0.456 0 0.001 0.002 0.004 0.009 0.012 0.015 0.026 0.022 0.014 0.018 0.026 0.015 0.022 0.023 0.022 0.021 0.016 0.014 0.015 0.013 0.016 0.016 0.017 0.012 0.012 0.014 0.013 0.016 0.014 0.01 0.01 0.009 0.009 0.066 ]	40	[ 0.425 0 0.002 0.002 0.004 0.007 0.018 0.015 0.009 0.023 0.021 0.034 0.036 0.01 0.017 0.021 0.015 0.018 0.014 0.012 0.007 0.015 0.014 0.016 0.016 0.014 0.011 0.016 0.01 0.011 0.011 0.009 0.009 0.013 0.005 0.007 0.005 0.011 0.005 0.092 ]	40	[ 0.425 0 0.002 0.002 0.004 0.007 0.019 0.016 0.01 0.024 0.019 0.035 0.035 0.01 0.013 0.021 0.015 0.019 0.015 0.011 0.008 0.016 0.013 0.017 0.017 0.013 0.009 0.017 0.009 0.012 0.011 0.008 0.008 0.014 0.006 0.008 0.006 0.011 0.004 0.091 ]

Diseases—names of dairy diseases; HMC30 Opt. number of states $R^{*}$—Optimal number of states calculated for Homogenous Markov chain model for next 30 days; HMC 30 Predicted Distribution—predicted probability distribution of optimal states for Homogenous Markov chain model for next 30 days; HMC 60 Opt. number of states $R^{*}$—Optimal number of states calculated for Homogenous Markov chain model for next 60 days; HMC 60 Predicted Distribution—predicted probability distribution of optimal states for Homogenous Markov chain model for next 60 days; NHMC 30 Opt. number of states $R^{*}$—Optimal number of states calculated for Non-Homogenous Markov chain model for next 30 days; NHMC 30 Predicted Distribution—predicted probability distribution of optimal states for Non-Homogenous Markov chain model for next 30 days; NHMC 60 Opt. number of states $R^{*}$—Optimal number of states calculated for Non-Homogenous Markov chain model for next 60 days; NHMC 60 Predicted Distribution—predicted probability distribution of optimal states for Non-Homogenous Markov chain model for next 60 days.
